# Additional value of screening for minor genes and copy number variants in hypertrophic cardiomyopathy

**DOI:** 10.1371/journal.pone.0181465

**Published:** 2017-08-03

**Authors:** Irene Mademont-Soler, Jesus Mates, Raquel Yotti, Maria Angeles Espinosa, Alexandra Pérez-Serra, Ana Isabel Fernandez-Avila, Monica Coll, Irene Méndez, Anna Iglesias, Bernat del Olmo, Helena Riuró, Sofía Cuenca, Catarina Allegue, Oscar Campuzano, Ferran Picó, Carles Ferrer-Costa, Patricia Álvarez, Sergio Castillo, Pablo Garcia-Pavia, Esther Gonzalez-Lopez, Laura Padron-Barthe, Aranzazu Díaz de Bustamante, María Teresa Darnaude, José Ignacio González-Hevia, Josep Brugada, Francisco Fernandez-Aviles, Ramon Brugada

**Affiliations:** 1 Cardiovascular Genetics Center, University of Girona-IDIBGI, Girona, Spain; 2 Centro de Investigación Biomédica en Red de Enfermedades Cardiovasculares (CIBERCV), Madrid, Spain; 3 Department of Cardiology, Hospital General Universitario Gregorio Marañón, Instituto de Investigación Sanitaria Gregorio Marañón. Universidad Complutense, Madrid, Spain; 4 Department of Medical Sciences, School of Medicine, University of Girona, Girona, Spain; 5 Gendiag.exe SL, Barcelona, Spain; 6 Inherited Cardiac Diseases Unit. Department of Cardiology. Hospital Universitario Puerta de Hierro, Francisco de Vitoria University, Madrid, Spain; 7 Genetics Unit, Hospital Universitario de Móstoles, Madrid, Spain; 8 Hospital Universitario Miguel Servet, Zaragoza, Spain; 9 Arrhythmia Unit, Hospital Clinic de Barcelona, University of Barcelona, Barcelona, Spain; 10 Cardiovascular Genetics Unit, Hospital Universitari Dr. Josep Trueta, Girona, Spain; Pennsylvania State University, UNITED STATES

## Abstract

**Introduction:**

Hypertrophic cardiomyopathy (HCM) is the most prevalent inherited heart disease. Next-generation sequencing (NGS) is the preferred genetic test, but the diagnostic value of screening for minor and candidate genes, and the role of copy number variants (CNVs) deserves further evaluation.

**Methods:**

Three hundred and eighty-seven consecutive unrelated patients with HCM were screened for genetic variants in the 5 most frequent genes (*MYBPC3*, *MYH7*, *TNNT2*, *TNNI3* and *TPM1*) using Sanger sequencing (N = 84) or NGS (N = 303). In the NGS cohort we analyzed 20 additional minor or candidate genes, and applied a proprietary bioinformatics algorithm for detecting CNVs. Additionally, the rate and classification of *TTN* variants in HCM were compared with 427 patients without structural heart disease.

**Results:**

The percentage of patients with pathogenic/likely pathogenic (P/LP) variants in the main genes was 33.3%, without significant differences between the Sanger sequencing and NGS cohorts. The screening for 20 additional genes revealed LP variants in *ACTC1*, *MYL2*, *MYL3*, *TNNC1*, *GLA and PRKAG2* in 12 patients. This approach resulted in more inconclusive tests (36.0% vs. 9.6%, p<0.001), mostly due to variants of unknown significance (VUS) in *TTN*. The detection rate of rare variants in *TTN* was not significantly different to that found in the group of patients without structural heart disease. In the NGS cohort, 4 patients (1.3%) had pathogenic CNVs: 2 deletions in *MYBPC3* and 2 deletions involving the complete coding region of *PLN*.

**Conclusions:**

A small percentage of HCM cases without point mutations in the 5 main genes are explained by P/LP variants in minor or candidate genes and CNVs. Screening for variants in *TTN* in HCM patients drastically increases the number of inconclusive tests, and shows a rate of VUS that is similar to patients without structural heart disease, suggesting that this gene should not be analyzed for clinical purposes in HCM.

## Introduction

Hypertrophic cardiomyopathy (HCM) is characterized by left ventricular hypertrophy with histologic features of cellular hypertrophy, myofibrillar disarray, and interstitial fibrosis. With a prevalence of 0.2% in the adult population, HCM is the most common inherited cardiac disease and a major cause of sudden cardiac death (SCD) in young people [1]. The disease has marked phenotypic variability, and clinical manifestations range from asymptomatic clinical course to severe heart failure and SCD. The identification of a disease-causing variant in a patient is crucial for diagnosis confirmation in borderline cases, early management of at-risk family members, genetic counseling and avoidance of unnecessary follow-up of non-carriers. The latter, besides doubtless clinical benefit, enables significant health-care costs saving [2–4]. For all these reasons, current guidelines recommend genetic testing in patients fulfilling diagnostic criteria for HCM, but the advantages of screening for genes without a definitive evidence of disease association versus more conservative approaches remain to be determined [5].

Overall, in patients fulfilling HCM diagnostic criteria, genetic testing leads to the identification of disease-causing genetic variants in 32–78.9% of cases, depending on the clinical characteristics of the patients, the number of genes studied, and the criteria used for variant classification [4, 6–19]. Most HCM cases are caused by mutations in genes that encode sarcomere proteins [19–21]. Among them, about 85% of pathogenic variants are found in *MYBPC3* and *MYH7*, 10% in cardiac troponin T (*TNNT2*) and troponin I (*TNNI3*), up to 2% in *TPM1*, and less than 3% in other sarcomere genes (*MYL2*, *MYL3*, *ACTC1* and *TNNC1*). For this reason, initial studies using Sanger sequencing in HCM recommended to focus on the 5 principal sarcomere genes [20]. Recent improvements in DNA-sequencing technologies offer the opportunity to screen for a larger number of genes in a time and cost-effective manner. However, this approach also results in an increase of the number of rare genetic variants of unknown significance (VUS), which may entail a clinical challenge.

The analysis of a predefined panel of HCM-related genes using Next-Generation Sequencing (NGS) technologies has emerged as the preferred genetic testing methodology for clinical purposes in HCM. This approach allows the additional screening for genes that have been previously proposed to cause a relatively small number of HCM cases (minor genes) and genes with a controversial role in the disease (candidate genes) [12, 19, 22]. Moreover, panels can easily include genes related to metabolic disorders that account for rare cases of unexplained left ventricular hypertrophy in adults (<5%) but whose identification is of great clinical relevance [5]. Finally, NGS enables the detection of alterations in the number of copies of large genomic regions, known as Copy Number Variants (CNVs). Recently, two large NGS series involving the screening for these variants in HCM-associated genes have shown that 0.56–0.8% of HCM cases may be explained by these large imbalances [23, 24].

The aim of the present study was to determine the prevalence and spectrum of clinically relevant genetic variants in a Spanish cohort of HCM patients and analyze the additional clinical value provided by the screening for minor and candidate HCM genes and CNVs using NGS. The value and clinical challenges derived from the screening for variants in *TTN* were specifically addressed and compared with an independent cohort of patients without structural heart disease.

## Materials and methods

### Study population

The study cohort includes 387 consecutive unrelated Spanish patients with clinical diagnosis of HCM according to current clinical criteria [5], referred for genetic testing between 2012 and 2016. The study was approved by the ethical committee of Hospital Universitari Dr. Josep Trueta de Girona (Spain) and conformed to the ethical guidelines of the Declaration of Helsinki 2008. Informed written consent was obtained from all subjects. Patients were recruited at the Inherited Heart Diseases Units from Hospital General Universitario Gregorio Marañón, Hospital Universitari Dr. Josep Trueta, Hospital Clínic de Barcelona and Hospital Universitario Puerta del Hierro. The mean age at the time of the genetic study was 48±20 years, 255 patients (65.9%) were men and 132 (34.1%) women. Family members of carriers of rare non-synonymous variants, indels and/or CNVs were invited to undergo genetic analysis. During the period of the study 180 relatives were referred for genetic testing and were studied by Sanger sequencing or MLPA.

The detection rate and classification of rare variants in *TTN* in the HCM cohort was compared with the results obtained in an independent group of 427 unrelated patients without echocardiographic evidence of structural heart disease (30 healthy subjects, 191 patients with Brugada syndrome, 138 with long QT syndrome, 20 with catecholaminergic polymorphic ventricular tachycardia, 8 with short QT syndrome, 9 with atrioventricular block, 7 with idiopathic ventricular tachycardia/ ventricular fibrillation, 5 with atrial fibrillation and 19 with other arrhythmias).

### Genetic analysis

Total genomic DNA was isolated from blood or saliva samples using Chemagen MSM I (PerkinElmer, Germany). All patients were screened for the 5 more frequent sarcomere genes (*MYBPC3*, *MYH7*, *TNNI3*, *TNNT2 and TPM1*) (isoforms analyzed are listed in S1 Table). The first 84 patients underwent Sanger sequencing of these 5 genes and the remaining 303 patients were studied using expanded NGS panels.

#### Sanger sequencing

The coding regions and exon-intron boundaries (±10bp) of the 5 main sarcomere genes were amplified by PCR and, after purification, the PCR products were directly sequenced in both directions using BigDye Terminator v3.1 Cycle Sequencing Kit (Applied Biosystems, TX, USA). Sequencing products were run on 3130XL Genetic Analyzer (Applied Biosystems) and analyzed by means of SeqScape Software v2.5 (Life Technologies, CA, USA).

#### NGS

Three hundred and three patients were analyzed with custom NGS panels (55 or 78 genes) including the coding regions and exon-intron boundaries (±10bp) of the most prevalent genes associated with inherited cardiac diseases (the 55-gene panel includes the UTR sequences of some genes). Coordinates of sequence data were based on UCSC human genome version hg19 (GRCh37). Both NGS panels were developed by Gendiag.exe S.L. and commercialized by Ferrer InCode as SudD inCode®.

Using either panel, we focused on the analysis of 25 genes previously associated or candidate for HCM. Reference sequence transcripts listed in S1 Table were analyzed. Only eight sarcomere genes definitively associated with HCM (*MYBPC3*, *MYH7*, *TNNI3*, *TNNT2*, *TPM1*, *ACTC1*, *MYL2 and MYL3*) and genes robustly associated with metabolic diseases that can mimic HCM (*GLA*, *LAMP2* and *PRKAG2*) were considered validated genes [22]. The additional 14 genes (*CSRP3*, *PLN*, *ACTN2*, *MYOZ2*, *MYH6*, *TNNC1*, *CAV3*, *JPH2*, *LDB3*, *RYR2*, *TCAP*, *VCL*, *PDLIM3* and *TTN)* were classified as candidate genes. Following the criteria described by Walsh et al. [22], these candidate genes have different levels of evidence for their association with HCM, with the exception of *PDLIM3* and *VCL* (no supporting evidence), and *RYR2* and *TTN* (evidence not analyzed). We analyzed separately the additional value and clinical challenges derived from the screening for variants in *TTN*, due to the limited evidence for its association with HCM and the high background variation of this gene.

Sample libraries were prepared following the SureSelect XT Target Enrichment System for Illumina Paired-End Sequencing Library protocol (Agilent Technologies, CA, USA). Indexed libraries were sequenced in ten-sample pools on a MiSeq platform (Illumina, CA, USA), with 2x75 bp reads length.

An algorithm developed by Gendiag.exe SL was used to process the FASTQ files to obtain clean BAM files for the subsequent analysis of both SNVs and indels. In brief, the processed raw reads obtained after sequencing were trimmed and mapped with BWA-MEM [25]. The output from mapping steps was joined and sorted, and only the uniquely and properly mapped read pairs were selected. Then the variant call was performed with SAMtools v.1.2 [26], together with an *ad hoc* developed script. Both the custom NGS gene panels and the bioinformatics algorithm used for the detection of SNVs and indels had been previously validated in our center, obtaining a sensitivity of 100% and a specificity of 99.5% (unpublished data). The identified SNVs and indels were annotated with dbSNP [27], Exome Sequencing Project (ESP) [28], 1000 Genomes Project [29], Exome Aggregation Consortium (ExAC) [30], Human Gene Mutation Database (HGMD) [31] and ClinVar [32]. Sanger sequencing was performed to sequence regions with coverage lower than 30X, as well as to validate the uncommon non-synonymous variants identified. Genetic variants were reported following the recommendations of the Human Genome Variation Society.

We used a bioinformatics algorithm developed in our laboratory to detect CNVs using NGS data. The approach focuses on capturing significant differences between the expected and observed normalized coverage for a given sample in every exon of the genes included in the NGS panels. Raw coverage is first normalized by the amount of DNA yielded for each sample in the run. Then the insert size and the low probe affinity bias for targeted regions with a too high or too low GC content (>75% and <45%, respectively) are corrected. Finally, the ratio between each sample and a built-in baseline is evaluated. If the ratio falls outside a signal-to-noise window and is greater or lower than the duplication or deletion cut-offs (0.45 and -0.8, respectively), the gain or loss is inferred. Each potential CNV was visually reviewed to discard possible false positives due to artefacts caused by samples with enrichment inconsistencies generated during the library preparation protocol. Sensitivity and specificity of the method were assessed in an independent cohort including 108 patients with different cardiovascular diseases (16 of them with known CNVs and the remaining without this type of rearrangements), and they were 100% and 90.7%, respectively (unpublished data).

Each CNV identified by NGS was validated by an alternative method (Multiplex Ligation-dependent Probe Amplification -MLPA- or quantitative PCR -qPCR-). MLPA analyses were performed using commercially available SALSA MLPA probemixes and following manufacturer’s instructions (MRC-Holland, Amsterdam, The Netherlands). After the multiplex PCR reaction, electrophoresis was performed using ABI3130XL Genetic Analyzer with LIZ500 size standard (both from Applied Biosystems), and results were analyzed using Coffalyser.Net (MRC-Holland). qPCR analyses were performed with the QuantStudio 7 Flex System using Power Up Sybr Green master mix (both from Life Technologies), following manufacturer’s recommendations. Results were analyzed with QuantStudio Real-Time PCR Software v1.2 (Life Technologies).

For precise characterization of the CNVs, the breakpoints were assessed using NGS split-read data when the breakpoint regions were covered by the NGS panels, and then they were confirmed by Sanger sequencing. When no split-read data were available, Sanger sequencing was performed in an attempt to characterize the breakpoints using primers located in the non-altered regions of the gene of interest.

#### Summary of NGS data results

In the present study, including all MiSeq runs, the average call rate achieved at 30x with the custom enrichment gene designs of 55 and 78 genes was 99.7% and 99.8%, respectively. The median percentage of reads overlapping our target regions was 48% (range 39% to 51%) for the first panel and 66% (range 52% to 69%) for the second one. The median coverage per sample was 870 (721 to 1069) and 679 (479 to 867), respectively. The 25 and 75 percentiles were 571 and 1099 for the 55 gene design, and 509 and 843 for the 78 gene design.

### Criteria for interpretation of SNVs, indels and CNVs

Rare variants (SNVs and indels) were defined as variants with a minor allele frequency (MAF) <0.002 [19, 33] in the databases dbSNP [27], ESP [28], 1000 Genomes Project [29] and Exome Aggregation Consortium (ExAC) [30]. We chose this conservative and inclusive cut-off to ensure the selection of all potentially relevant variants for the subsequent process of individual variant classification (see below). Additionally, to analyze the impact of the MAF filter applied on the final number and classification of genetic variants, we compared this approach with a more restrictive hard filtering recently proposed by Walsh et al., based on the frequency of the most common pathogenic HCM variant (MAF <0.0001 in ExAC) [22]. Both MAF criteria were also used to compare the detection rate of rare variants in *TTN* in HCM patients and individuals without a structural heart disease.

We used the updated American College of Medical Genetics and Genomics (ACMG) 2015 guidelines for variant interpretation to classify variants in 5 categories: pathogenic (P), likely pathogenic (LP), VUS, likely benign (LB) or benign (B) [34]. For the assessment of the clinical significance of previously reported variants we first searched for information in public variant databases, population cohorts and scientific literature. Then, available clinical, experimental and computational data were integrated with potential additional information obtained from the study of the particular family to reach a final clinical conclusion. The strength of the association with the disease at the gene-level was classified as strong, moderate, weak, only supported in functional data or no evidence [22].

Novel variants were defined as variants not previously reported in patients (published literature, HGMD [31] or ClinVar [32]) and absent from controls in ESP, 1000 Genomes Project [29], ExAC and Genome Aggregate Database [30]. Novel variants that did not meet strict ACMG criteria of pathogenicity (VUS) but exhibited at least one supportive criteria were denominated novel candidate variants. For this purpose we considered the following criteria: 1) location in a mutational hot spot and/or critical and well-established functional domain, 2) protein length changes as a result of in-frame deletions/insertions in a nonrepeat region, 3) missense change at an amino acid residue where a different missense change determined to be pathogenic has been seen before, 4) cosegregation with disease in ≥2 affected family members, or 5) multiple lines of computational evidence support a deleterious effect (probably or possibly damaging/deleterious/disease causing by three *in silico* prediction tools: PolyPhen-2 [35], Provean [36] and Mutation Taster [37]).

Finally, if the segregation study of a large family enabled the classification of a variant as LB (no segregation), such labeling was extrapolated to other index cases with the same genetic variant. In the description of *TTN* variants, we included their location in the main protein domains and the percentage spliced in (PSI) of the affected exon (estimation of the percentage of *TTN* transcripts that incorporate the mutation) [38], but we did not use this information to modify the variant classification.

Confirmed CNVs were first compared with published literature and databases HGMD [31], ClinVar [32], DECIPHER [39], Database of Genomic Variants [40] and ClinGen [41]. If the CNV (identical after precise characterization) had been previously robustly classified as P/LP or B/LB, the classification was extrapolated to our case. Novel CNVs were classified as pathogenic variants if: 1) it was a deletion in/of a gene where loss of function is a known mechanism of patient’s disease, 2) it was an intragenic in tandem duplication (not involving the last exon of the gene) in a gene where loss of function is a known mechanism of disease, or 3) it was a whole gene duplication in a gene for which triplosensitivity is known to cause patient’s disease.

### Statistical analysis

Categorical variables were compared using the chi-square test and two-sided p values <0.05 were considered significant. Specifically, we compared the percentage of patients with rare variants observed after the screening for 25 genes with the percentage obtained when only the 5 main sarcomere genes were analyzed in the same cohort. We also compared the rate of P/LP variants found using these two different approaches. The same comparison was performed excluding *TTN* (set of 24 genes). Contingence tables were built to identify the number of patients with rare variants in *TTN* that also carried rare or P/LP variants in sarcomere genes. The role of the MAF hard filter on the final number and classification of genetic variants was analyzed comparing the proportions obtained using two different MAFs (<0.002 vs. <0.0001). We also used both MAF cut-offs to compare the detection rate of rare variants in *TTN* in patients with HCM and patients without structural heart disease. The statistical analysis was performed using R version 3.3.2.

## Results

### Genetic variants in main sarcomere genes

Overall, including both the Sanger sequencing and the NGS cohorts (n = 387 patients), we found 187 rare variants in the 5 principal sarcomere genes (*MYBPC3*, *MYH7*, *TNNI3*, *TNNT2* and *TPM1*) in 269 patients (69.5%) (S1 and S2 Tables). After applying the ACMG criteria of causality, 135 variants were classified as P/LP (72.2%), 41 (21.9%) were considered VUS, and only 11 (5.9%) were LB. No significant differences in the percentage of rare, P/LP or novel variants in the five main sarcomere genes were observed between the Sanger and the NGS cohorts (split data are shown in Table 1). The percentage of patients with at least one P/LP variant (positive test) was 33.3%. We found 48 novel variants in these sarcomere genes. Among them, 35 were classified as P/LP (Table 2) and 1 as candidate novel variant.

**Table 1:** Rare variants (MAF <0.002) in the 5 most frequent sarcomere genes, 25 genes associated with or candidate for HCM and 24 genes (same panel excluding *TTN*).

	Main Sarcomere Genes			25 Gene Panel	24 Genes (excluding *TTN*)
	Pooled Data	Sanger cohort	NGS cohort	NGS cohort	NGS cohort
**Patients n**	**387**	**84**	**303**	**303**	**303**
Positive test n (%)	129 (33.3)	30 (35.7)	99 (32.7)	111 (36.6)	111 (36.6)
Positive test or novel candidates n (%)	130 (33.6)	30 (35.7)	100 (33.0)	121 (39.9)	115 (38.0)
Test with non-bening variants^(1)^ n (%)	163 (42.1)	35 (41.7)	128 (42.2)	220 (72.6)*	171 (56.4)*^#^
Inconclusive test n (%)	34 (8.8)	5 (6.0)	29 (9.6)	109 (36.0)*	60 (19.8)*^#^
**Number of rare variants**	**187**	**39**	**148**	**398**	235*^#^
Pathogenic n (%)	68 (36.4)	14 (35.9)	54 (36.5)	57 (14.3)*	57 (24.3)*^#^
Likely Pathogenic n (%)	67 (35.8)	16 (41.0)	51 (34.5)	60 (15.1)*	60 (25.5)^#^
VUS n (%)	41 (21.9)	6 (15.4)	35 (23.6)	243 (61.1)*	97 (41.3)*^#^
Benign/Likely Benign n (%)	11 (5.9)	3 (7.7)	8 (5.4)	38 (9.5)	21 (8.9)
**Novel variants**	**48**	**10**	**38**	110*	**61**^#^
Pathogenic/Likely Pathogenic n (%)	35 (72.9)	9 (90)	26 (68.4)	29 (26.4)*	29 (47.5)^#^
Novel Candidate variants	1 (2.1)	0	1 (2.6)	27 (24.5)*	7 (11.5)^#^
VUS (excluding candidate variants) n (%)	11 (22.9)	1 (10)	10 (26.3)	51 (46.4)*	24 (39.3)
Benign/Likely Benign n (%)	1 (2.1)	0	1 (2.6)	3 (2.7)	1 (1.6)

(1) All rare variants excluding benign and likely benign variants. n: number; NGS: next generation sequencing; VUS: variant of unknown significant.

*p<0.05 vs. analysis of 5 genes (*MYBPC3*, *MYH7*, *TNNI3*, *TNNT2* and *TPM1*) in the NGS cohort.

^#^p<0.05 vs. panel including 25 genes.

**Table 2:** Novel pathogenic/likely pathogenic variants found in validated sarcomere genes.

Gene	cDNA	Aminoacid	Exon	Type	Probands
***MYBPC3***					
	c.323delC	p.108Lfs*51	3	Frameshift	1
	c.313dupG	p.A105Gfs*8	3	Frameshift	1
	c.572G>T	p.W191L	5	Missense	1
	c.1421_1424delAGTG	p.E474Vfs*13	16	Frameshift	1
	c.1471delG	p.V491Wfs*3	17	Frameshift	1
	c.2190delC	p.K731Rfs*23	23	Frameshift	2
	c.2329dupG	p.A777Gfs*56	24	Frameshift	2
	c.2591delT	p.F864Sfs*15	25	Frameshift	1
	c.2512G>T	p.E838*	25	Nonsense	1
	c.2724_2725delCTinsGCTGTA	p.Y908*	26	Nonsense	1
	c.2603-2A>G		26	Splice site	2
	c.2905+2T>C		27	Splice site	1
	c.3066dupC	p.N1023Qfs*28	29	Frameshift	2
	c.3190+5G>C		29	Intronic	1
	c.3182_3190+4delAGGTTGTTGGTGC		29	Long indel	1
	c.3020G>A	p.W1007*	29	Nonsense	2
	c.3190+2T>C		29	Splice site	1
	c.3328delA	p.M1110Wfs*79	30	Frameshift	3
	c.3620_3623dupGCCC	p.K1209Pfs*34	32	Frameshift	1
	c.3719T>A	p.I1240N	33	Missense	1
***MYH7***					
	c.530C>G	p.T177S	6	Missense	1
	c.920C>G	p.P307R	11	Missense	2
	c.1207C>G	p.R403G	13	Missense	1
	c.1580C>T	p.P527L	16	Missense	1
	c.2596T>C	p.S866P	22	Missense	1
***TNNT2***					
	c.311C>A	p.A104E	9	Missense	1
***TNNI3***					
	c.602T>C	p.M201T	8	Missense	1

**Fig 1:**
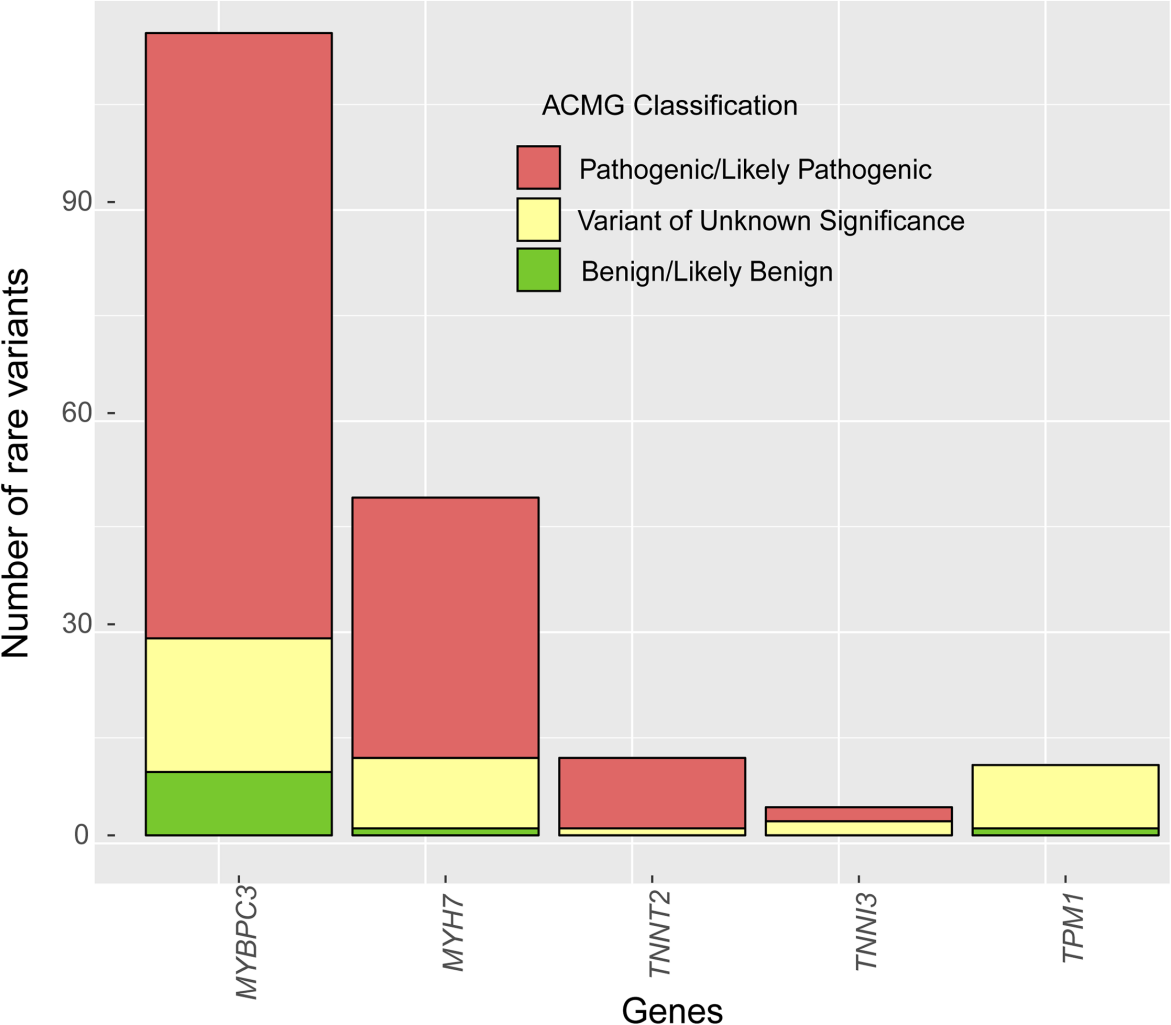
Classification of rare variants in *MYBPC3*, *MYH7*, *TNNI3*, *TNNT2* and *TPM1* (pooled data from Sanger sequencing and NGS cohorts).

The distribution of the 187 rare variants found in the 5 main sarcomere genes (pooled data from Sanger sequencing and NGS cohort, 387 patients) and their clinical classification is shown in Fig 1. We found 114 rare variants in *MYBPC3*, 48 in *MYH7*, 11 in *TNNT2*, 4 in *TNNI3* and 10 in *TPM1*. As expected, most P/LP variants were found in *MYBPC3* (64.2%) and *MYH7* (27%). In accordance with a well-known loss of function mechanism for *MYBPC3*, most P/LP variants in this gene (60.2%) were radical variants, whereas missense variants were the most frequent variants among the other genes. Cascade genetic screening allowed us to establish the penetrance of 40 P/LP variants: 17 with complete penetrance and 23 with incomplete penetrance. One variant was *de novo*.

We identified several sarcomere variants previously associated with other cardiomyopathies (S2 Table). Among P/LP variants, *MYH7*_p.R249G has been previous described in association with left ventricular non-compaction cardiomyopathy (LVNC) [42], and *MYH7*_p.T1019N in association with both dilated cardiomyopathy (DCM) and HCM [43, 44]. Additionally, cascade screening for the pathogenic variant *TNNT2*_p.E163del found in one HCM index case showed that 1 of the 3 genotype-positive relatives had HCM but the other 2 had a LVNC phenotype.

### Rare genetic variants identified in the NGS cohort

**Fig 2:**
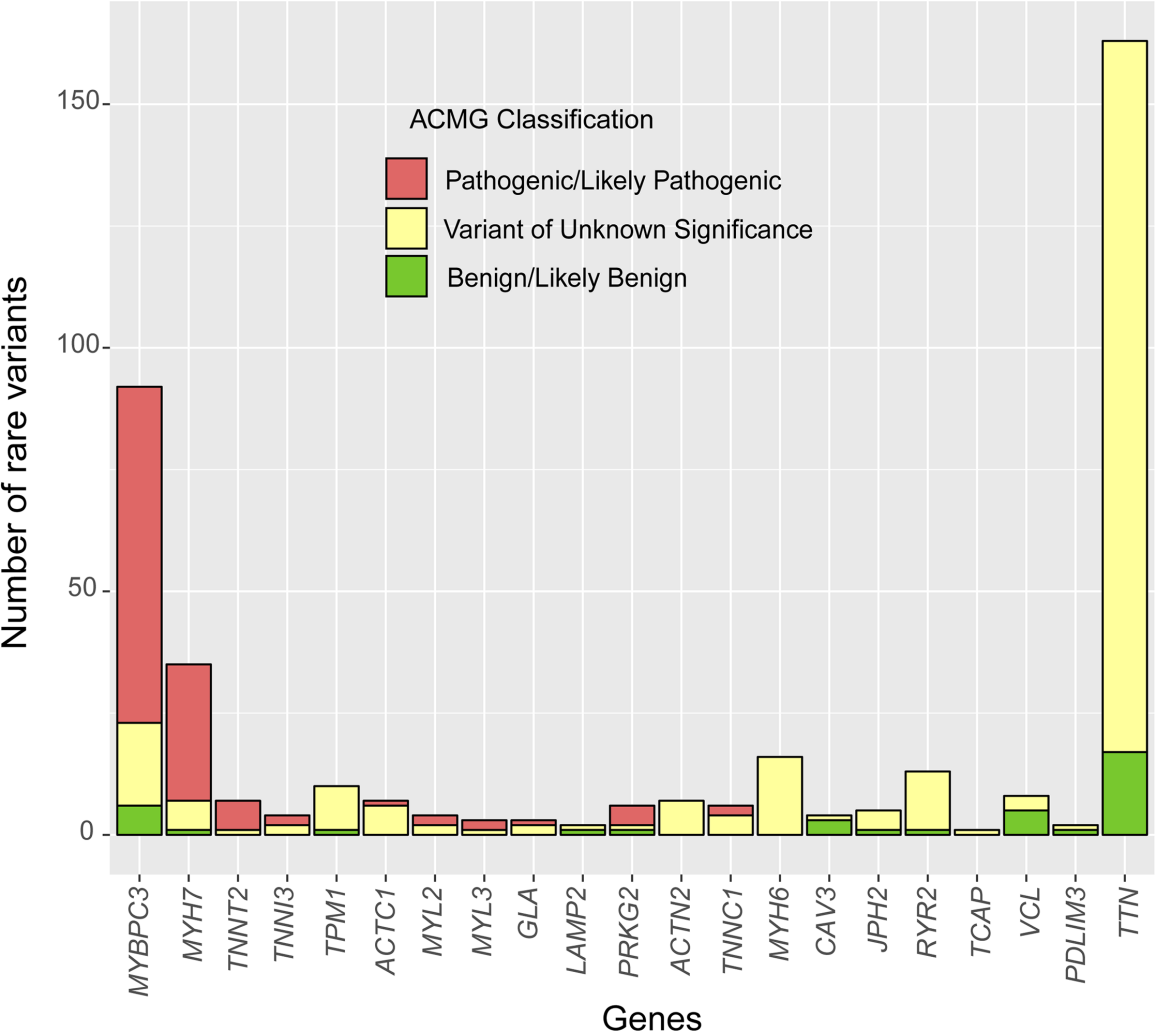
Classification of the rare variants found in the 25 genes screened in the NGS cohort.

**Fig 3:**
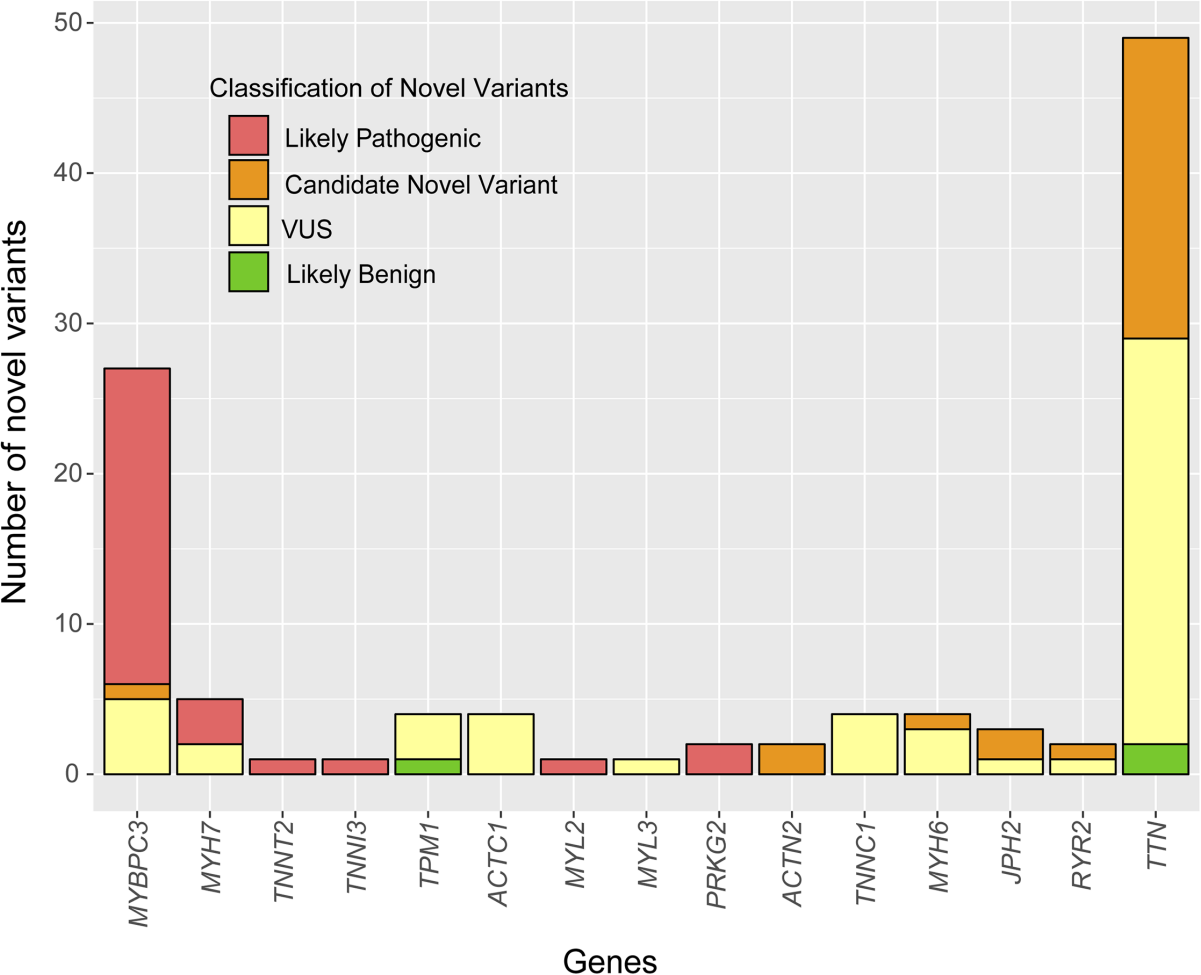
Classification of the novel variants identified in the NGS cohort.

The screening for 25 genes in the NGS cohort showed 401 rare variants, but 3 variants were not confirmed by Sanger sequencing (false discovery rate = 0.75%). Therefore, 398 confirmed variants were reported in 231 patients. In comparison with the screening for only the 5 main sarcomere genes, the percentage of patients with non-benign variants was significantly higher (72.6% vs. 42.2%, p<0.001). Among them, 117 variants in 111 patients (36.6%) were classified as P/LP (Table 1). The proportion of patients with a positive test was not significantly different to the proportion found when screening for only the 5 main sarcomere genes (Table 1). The distribution of the 398 rare variants in the 25 genes in the NGS cohort and their clinical classification is shown in Fig 2. All genes except *CSRP3*, *LDB3*, *MYOZ2* and *PLN* showed at least one rare variant. The classification of the novel variants identified in this cohort is shown in Fig 3.

**Fig 4:**
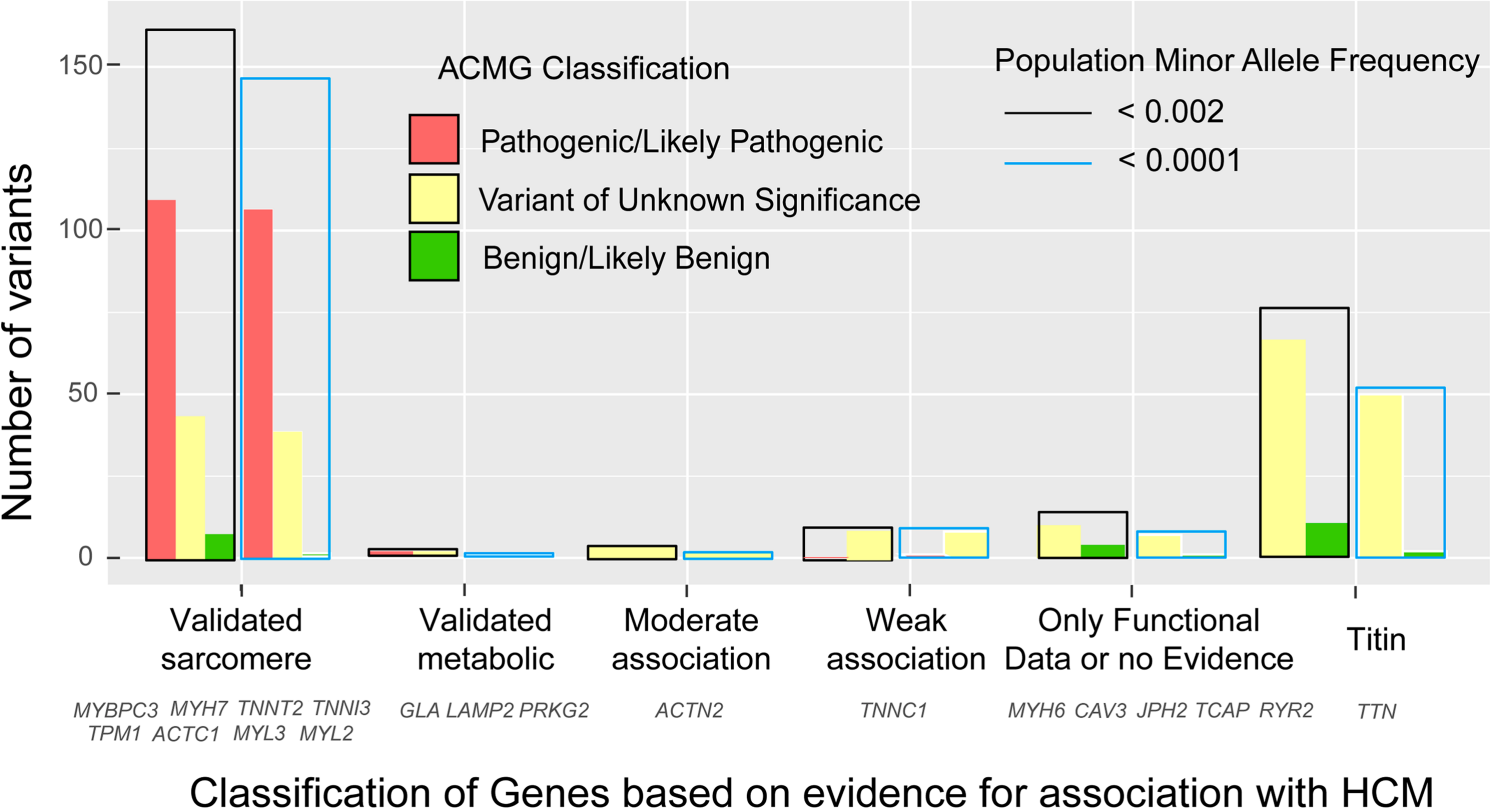
Distribution of rare variants according to gene-level supporting evidence, ACMG clinical classification and minor allele frequency filtering.

In 99 patients who did not have rare variants in the 5 main sarcomere genes the screening for minor and candidate genes identified 250 rare variants. Among them, 12 patients carried P/LP variants. Most of these P/LP variants were found in validated minor sarcomere genes (*ACTC1*, *MYL2* and *MYL3*) and genes related to metabolic diseases (*GLA* and *PRKAG2*) (Figs 2 and 4). The lack of definitive association with HCM implied that most rare variants found in candidate genes did not meet enough standardized ACMG criteria to be considered P/LP. However, at the individual variant level, we identified 2 unrelated HCM patients with the variant *TNNC1*_p.A8V, which has been previously reported in at least 7 additional unrelated HCM patients, is absent in control population (ExAC), and has functional studies supportive of a damaging effect. The findings of our cohort may help to increase the supportive evidence for enrichment of this infrequent variant in HCM cases.

The expanded genetic study identified 72 novel variants out of the 5 more frequent genes. On the other hand, the number of VUS drastically increased with the screening of 25 genes (23.6% vs. 61.1%, p<0.001). Most of the additional VUS were *TTN* variants (97.9% of them missense). Accordingly, the number of inconclusive tests also increased (9.6% vs. 36.0%, p<0.001) (Table 1).

#### Effect of MAF cut-off on the number and classification of rare variants

Using a ExAC MAF <0.0001 we identified 308 variants in 205 patients (67.7%) from the NGS cohort. This cut-off filtered out 28 of the 39 LB variants and 59 of the 241 VUS with MAF<0.002. However, the rate of inconclusive studies did not significantly change (31.7% vs. 36.0%, p = 0.26). Three LP variants in 3 different patients were missed (*MYBPC3*_p.V771M in two patients and *TNNT2_*p.R278C in another one). Although there is enough supporting evidence to consider both variants as LP, published data suggest incomplete penetrance and a mild effect in isolation. Complete analysis with MAF <0.0001 is provided in S3 Table. The effect of the different MAF cut-offs on the number and distribution of variants according to the gene-level association with the disease is presented in Fig 4.

#### Identification of multiple variants: Compound and double heterozygotes

Overall, considering only the 8 validated sarcomere genes and excluding LB variants, 14 patients carried two variants. In 6 cases we found 2 P/LP variants, and in 8 cases one P/LP variant in combination with one VUS. Six patients had two variants in *MYBPC3* (1 was a compound heterozygote, while in the remaining cases we cannot discard the possibility of both variants being located in the same allele), 1 patient had two variants in *MYH7*, and 7 patients were double-heterozygous for validated sarcomere genes. In the NGS cohort, 105 patients had more than one non-benign variant. After excluding *TTN* from analysis, 38 patients had multiple non-benign variants.

### Detection rate of *TTN* variants in HCM and comparison with patients without structural heart disease

#### *TTN* variant classification and filtering

We found 163 rare variants in *TTN* in 117 patients, 49 novel and 114 previously described in clinical and/or population databases. After a careful revision of published data and family studies, 17 variants were classified as LB (15 previously reported and 2 novel variants). In particular, segregation studies allowed us to classify the variant as LB in 8 families. The remaining 146 variants in 108 patients were classified as VUS (28 patients had 2 additional VUS, and 5 had 3 VUS). Fifty-eight patients also carried variants in the main sarcomere genes, classified as P/LP in 40 cases. In an attempt to select the VUS in *TTN* with a higher potential clinical significance we focused on 20 novel candidate variants found in 18 patients in which three *in silico* tools consistently predicted a deleterious effect (Table 3). Fourteen of these variants were found in 13 patients who also carried variants in sarcomere genes (9 patients had a P/LP variant and 4 a VUS). Only 6 of these filtered *TTN* variants in 5 patients were found in cases without variants in the main sarcomere genes.

**Table 3:** Novel variants of unknown significance in *TTN* gene that are deleterious according to multiple in silico predictors.

Patient ID	cDNA	Aminoacid	Exon	PSI	Domain	Variants in sarcomere genes in the same patient^(1)^
44	c.78293C>T	p.T26098I	275	100	A-band	Pathogenic: *MYBPC3* c.1505G>A p.R502Q
52	c.62924A>T	p.D20975V	275	100	A-band	Pathogenic: *MYBPC3* c.1624G>C p.E542Q
53	c.81646G>T	p.V27216F	283	100	A-band	Likely pathogenic: *MYBPC3* c.2724_2725delCTinsGCTGTA p.Y908*
60	c.89236A>G	p.K29746E	297	100	A-band	VUS Novel: *MYBPC3* c.631G>A p.D211N
**66**	c.85081G>A	p.A28361T	288	100	A-band	Pathogenic: *MYBPC3* c.162delG K54Nfs*13
**66**	c.93829T>C	p.Y31277H	307	100	M-band	Pathogenic: *MYBPC3* c.162delG K54Nfs*13
71	c.78293C>T	p.T26098I	275	100	A-band	Pathogenic: *ACTC1* c.889G>T p.A297S
95	c.8920A>G	p.M2974V	38	100	I-band	Likely Pathogenic: *MYH7* c.2608C>T p.R870C
**98**	c.758C>T	p.T253I	6	100	Z-disc	None
**98**	c.70579C>G	p.P23527A	275	100	A-band	None
101	c.51661G>A	p.D17221N	250	100	A-band	VUS Novel: *TNNC1* c.121C>A p.L41M
104	c.40364C>T	p.S13455F	205	100	A-band	None
108	c.46801C>A	p.P15601T	231	100	A-band	VUS Novel: *TPM1* c.632C>T p.A211V
112	c.90118A>G	p.R30040G	300	100	A-band	None
146	c.89159C>G	p.P29720R	296	100	A-band	None
170	c.16069C>T	p.P5357S	65	6	I-band	Likely pathogenic: *TNNT2* c.857G>A p.R286H
189	c.72563G>C	p.R24188T	275	100	A-band	Likely pathogenic: *MYL3* c.427G>A p.E143K
217	c.72098G>C	p.G24033A	275	100	A-band	Pathogenic: *MYBPC3* c.2308G>A p.D770N
245	c.37807G>C	p.G12603R	195	100	I-band	VUS: *TNNI3* c.304G>A p.A102T
260	c.47179C>T	p.P15727S	232	100	A-band	None

PSI: percent of splice in.

In bold: patients with 2 different novel variants in *TTN*.

^(1)^ additional information available in S2 Table.

#### Comparison with patients without structural heart disease

The number of HCM patients with non-synonymous rare variants in *TTN* (38.6% with MAF <0.002 and 29% with MAF <0.0001) was not significantly different to the group of patients without evidence of structural heart disease (39.3% with MAF <0.002, and 26% with MAF <0.0001) (p >0.3 for both case-control comparisons) (Table 4). The detection rate of VUS and novel variants was also similar (Table 4). A detailed description of the *TTN* variants analyzed in this cohort is provided in S4 Table.

**Table 4:** Detection rate and classification of variants in *TTN* in patients with hypertrophic cardiomyopathy and patients without structural heart disease.

	HCM (N = 303)		Non-structural (N = 427)	
	MAF <0.002	MAF <0.0001	MAF <0.002	MAF <0.0001
**Patients**				
with Rare Variants in *TTN*	117 (38.6)	88 (29.0)	168 (39.3)	111 (26.0)
with VUS in *TTN*	108 (35.6)	86 (28.4)	146 (34.2)	111 (26.0)
with Novel Variants in *TTN*	42 (13.9)	42(13.9)	50 (11.7)	50 (11.7)
***TTN* variants**	163	109	274	151
Likely Benign	17 (10.4)	3 (2.8)	63 (23.0)*	0 (0.0)
VUS	146 (89.6)	106 (97.2)	211 (77.0)*	151 (100)*
Novel	49 (30.1)	49 (45.0)	58 (21.2)*	58 (38.4)
Novel VUS deleterious *in silico*	20 (12.3)	20 (18.3)	17 (6.2)*	17 (11.2)
Truncating variants^(1)^	0 (0.0)	0 (0.0)	3 (1.1)	3 (2.0)
In consitutive Exons (PSI = 100)	123 (75.5)	81 (74.3)	230 (83.9)*	124 (82.1)
**Location in protein**				
A band	97 (59.5)	66 (60.6)	190 (69.3)*	98 (64.9)
I band	45 (27.6)	32 (29.4)	64 (23.4)	42 (27.8)
M band	13 (8)	8 (7.3)	17 (6.2)	10 (6.6)
Z disk	8 (4.9)	3 (2.8)	3 (1.1)*	1 (0.7)

HCM: hypertrophic cardiomyopathy; MAF: minor allele frequency in ExAC; PSI: percent of splice in; VUS: Variant of unknown significance.

^(1)^ Truncating: Nonsense, frameshift or canonical splicing.

* p<0.05 non-structural vs. HCM using the same MAF filter.

With respect to the total number of *TTN* variants, filtering with MAF <0.002, the percentage of LB variants was higher in the group of patients without structural heart disease, and the relative percentage of novel variants was lower. However, considering only variants with MAF <0.0001, the percentage of novel variants was not significantly different. The location of the variants in the protein and the percentage of variants located in constitutive exons (PSI = 100) were similar in both groups when using MAF <0.0001 (Table 4). Applying the same step-wise algorithm in the non-structural cohort we found 17 novel VUS consistently predicted as deleterious, 3 of them non-sense variants located in the A-band, I-band and M-band, respectively (S4 Table). The detection rate of patients with this type of selected novel variants was not significantly different between HCM patients and patients without structural heart disease.

### Identification, characterization and classification of CNVs

Screening for CNVs in our NGS cohort revealed that 4 out of the 303 patients had a validated CNV in one of the 25 genes analyzed (1.3%). Twelve additional signals were detected but they were not validated by MLPA or qPCR (false discovery rate = 75%). Among confirmed CNVs, 2 patients had deletions involving *MYBPC3* gene, and 2 patients had a deletion of the entire coding region of the *PLN* gene. According to our criteria for interpretation of CNVs, all of them were considered pathogenic variants.

#### CNVs in *MYBPC3*

**Fig 5:**
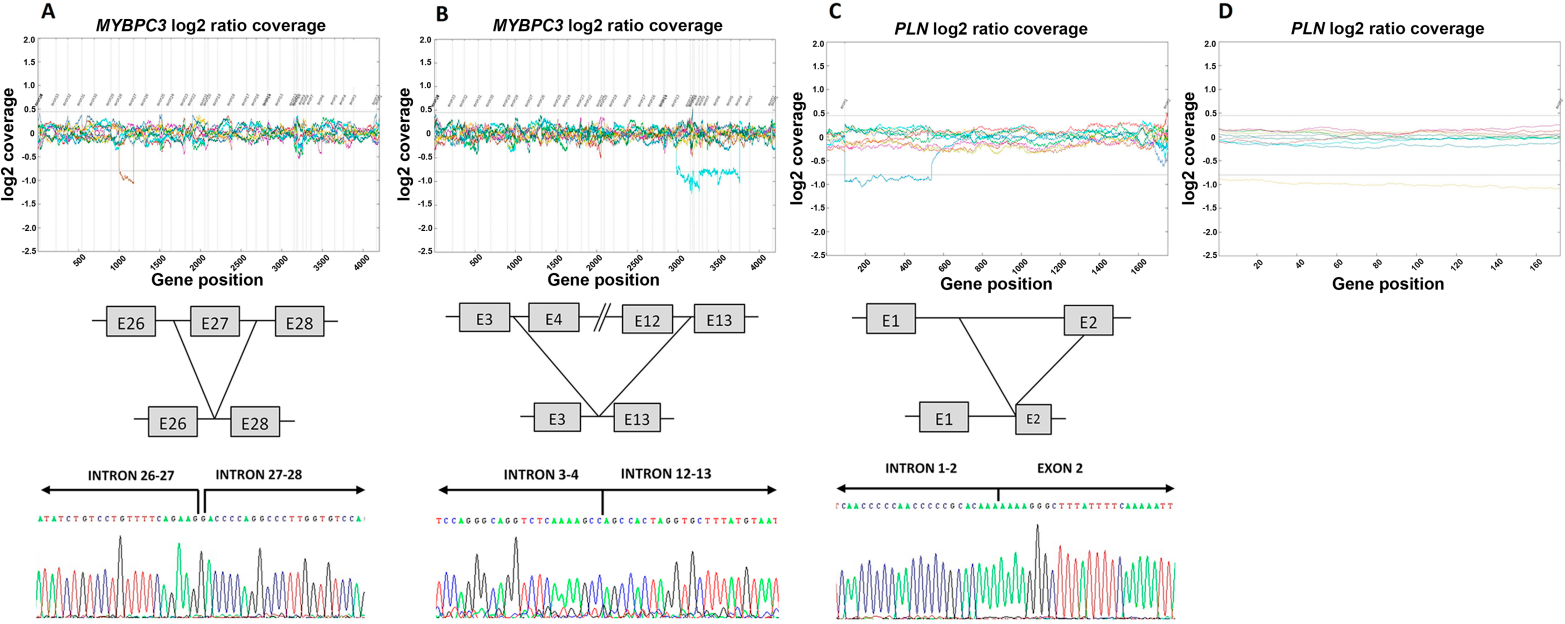
Cases with confirmed CNVs. NGS results, schematic representation of the breakpoints and precise characterization by Sanger sequencing of (**A**) the deletion of exon 27 of *MYBPC3* (P168, brown sample in the graph), (**B)** the deletion spanning from exon 4 to exon 12 of *MYBPC3* (P259, turquoise sample in the graph), and (**C**) the well-characterized *PLN* deletion (blue sample in the graph). (**D**) NGS results are shown for the non-characterized *PLN* deletion (orange sample in the graph).

One case (P168) had a deletion of the entire exon 27 (Fig 5. Panel A), and the other one (P259) had a deletion spanning from exon 4 to exon 12 (Fig 5. Panel B). Both deletions were confirmed by MLPA. No split-read data were available for the establishment of the rearrangement breakpoints of these cases, but both CNVs could be precisely characterized by Sanger sequencing: c.2737+148_2905+40del727insG for P168 and c.406+69_1091-1154del5654 for P259. None of the deletions has been previously described.

The case P168 also carried two previously reported variants in *MYBPC3* (p.V771M –classified as LP–and p.A522T –classified as LB–), and a VUS in *TTN*. The case P259 also carried two missense variants in *TTN*: p.E2055K (classified as LB) and p.R25906C (classified as VUS). Cascade genetic testing in this family showed that the deletion from exon 4 to exon 12 cosegregated with the disease in one affected relative; this patient also carried the variant *TTN_*p.R25906C but not the variant *TTN*_p.E2055K (S2 Table).

#### CNVs in *PLN*

In both cases, the deletions involved the entire coding region of the PLN gene and were confirmed by qPCR using the pair of primers 5’CTCAACAAGCACGTCAAAAGC3’ and 5’GCATCACGATGATACAGATCAGC3’. None of the patients had any uncommon SNP or indel. The first patient had a deletion of 7936bp, involving a portion of the first intron and the second exon of the gene (Fig 5. Panel C). The deletion was identified with the 55-gene panel, which included the UTR regions for this gene. The breakpoints could be identified using NGS split-read data, and were confirmed by Sanger sequencing. The precise description of the rearrangement was c.1-7587_159+190del7936. Such rearrangement has not been previously described. The second deletion of PLN was detected with the 78-gene panel, which does not include the UTR regions (Fig 5. Panel D). The rearrangement could not be further characterized using neither split-read data nor Sanger sequencing, but most probably is different from the one diagnosed in the other patient, as widening the coding region coordinates 500bp upstream and downstream the deletion signal was still present, and Sanger sequencing with the same pair of primers did not amplify any fragment. Overlapping rearrangements have not been previously described in HGMD, but there are several overlapping deletions described in DGV [40], DECIPHER [39], ClinGen [41] and ClinVar [32]. The variant found in DGV is a deletion of 58Kb involving the genes CEP85L and PLN, and the variants found in the other databases are larger and have been found in patients with congenital abnormalities.

## Discussion

In this study, we report the results of the genetic screening of 387 unrelated Spanish patients clinically diagnosed with HCM. Using NGS, we focus on the additional diagnostic value of screening for minor and candidate genes for HCM, and expose a comprehensive study of CNVs. Our data show that screening for these genes and CNVs in HCM patients identifies the genetic cause of the disease in a small number of cases, but this approach does not increase the global detection rate. The screening for variants in *TTN* in HCM patients shows a high number of VUS and increases the rate of inconclusive test. In an independent cohort without structural heart disease, we have found a number and classification of rare variants in *TTN* similar to that found in HCM patients; adding evidence against the role of this gene in HCM.

### Detection rate and clinical classification of rare variants

In the present study, the analysis of the most frequent sarcomere genes (*MYBPC3*, *MYH7*, *TNNI3*, *TNNT2* and *TPM1*) in patients with HCM identified a potentially relevant variant in 42.2% of the patients. Applying recommended criteria for clinical classification, P/LP variants were identified in only one third of the patients. The screening for additional sarcomere genes and other known and putative HCM genes using a 25-gene NGS panel showed potentially relevant variants in about two thirds of the patients, but identified an additional P/LP mutation in a small number of patients, without significantly increasing the percentage of patients with a positive test. Although some initial studies using Sanger sequencing reported a detection rate of pathogenic variants of 63% [7, 20], most studies have reported rates below 50% [6, 8, 9, 11, 13–18]. The detection rate in recent NGS studies including different additional genes range from 32% to 78.9% [4, 12, 17, 19, 44–52]. This huge variation is most likely due to selection bias in these studies, differences in the clinical characteristics of the patients included and, importantly, the different criteria applied for the classification of genetic variants. As extensively recognized, NGS offers a high reliability [44, 47], so the sequencing process itself does not justify the differences in the reported yields.

Interestingly, the percentage of patients with P/LP variants found in our study is in agreement with the largest published NGS series in patients with HCM, which includes more than 2900 unrelated HCM patients (with genetic screening involving from 10 to 51 known or putative HCM genes) [4]. The criteria for the assessment of genetic variants used in their study was similar to the ACMG classification followed in the present study. It has been recognized that this strict classification may result in a larger proportion of variants being considered VUS [34], but the main purpose of this tool is to guide clinical decisions, which must be always based on strong supporting evidence. Nevertheless, we recognize that these restrictive clinical classifications do not fulfill the requirements of a research study to identify new disease-causing variants, especially when genes with a non-definitive association with the disease are included. For this reason, an additional effort to weight the evidence of pathogenicity during the assessment of novel candidate variants identified by NGS should be attempted.

#### Genetic spectrum of the disease

In agreement with most series [4, 7–9, 14, 15, 17], our study shows that *MYBPC3* is the gene with a higher proportion of P/LP variants, followed by *MYH7*. While *MYH7* variants are almost exclusively missense (in our cohort all of them), *MYBPC3* is characterized by a significant incidence of radical variants [3, 4]. Overall, considering only the 8 validated sarcomere genes, the 3.6% of our patients carried two non-benign variants, which is consistent with published data [17, 20]. The screening for additional candidate genes using NGS increased the proportion of carriers of multiple non-benign variants to 34.7%, mostly due to the existence of rare missense *TTN* variants. It has been demonstrated that the presence of multiple pathogenic variants in the 8 validated sarcomere genes may confer a more severe form of disease with a higher incidence of adverse outcomes including heart failure and sudden death [53]. However, the clinical significance of the presence of multiple variants in the remaining genes is unknown and this information should not currently be used for prognostic purposes.

### Variants of unknown significance and role of *TTN*

While the screening for 25 genes provides a definitive diagnostic in particular cases without P/LP variants in the main sarcomere genes, the proportion of cases with VUS increases exponentially. These VUS represent nowadays a major clinical challenge, as proper genetic diagnosis and genetic counseling cannot be provided. In the assessment of VUS, the study of large pedigrees for segregation analyses and *in vitro* assays may provide useful information, but these studies are not possible or feasible in most cases.

Current available data are not enough to support pathogenicity of novel variants in candidate genes, but the absence in controls and the existence of consistent computational data supporting a deleterious effect should be taken into consideration to undertake segregation and/or functional studies. It is important to underlie that variants in candidate genes should not be used for clinical purposes, such as genetic counseling or cascade screening testing. However, reporting and carefully addressing them are necessary steps for the improvement of genetic diagnosis in HCM. Additionally, scientific literature and databases should be periodically searched for new information to reclassify these variants.

In the present study, the drastic increase of VUS in the NGS cohort is mainly due to the analysis of *TTN*, which is the gene with the largest coding sequence in the human genome. Whereas the pathogenic role of truncating variants in *TTN* has been demonstrated for DCM [54], the frequency of these radical variants in patients with HCM is similar to that found in control populations [54], and the pathogenic role of *TTN* missense variants is unknown [55]. In the present study we show that the number and classification of missense variants in *TTN* is not significantly different in patients with HCM and patients without structural heart disease. Even the rate of occurrence of novel variants that are consistently predicted as deleterious by *in silico* tools is not significantly different between both cohorts. Moreover, whereas a potential modifier role of selected missense *TTN* variants cannot be definitively ruled out, the finding of a high proportion of rare *TTN* variants in patients with variants in sarcomere genes increases the concerns about their actual pathogenic role. Altogether, these data may advice against the inclusion of this gene in clinical HCM panels.

### Role of CNVs in HCM

To date, the role of CNVs in patients with HCM is a relatively unexplored field. The first CNV in a patient with HCM was reported in 1992 and consisted of a 2.4Kb deletion in *MYH7*, which was identified by Southern blotting, analyzing restriction fragment length polymorphisms [56]. A second patient with HCM and two CNVs in *MYBPC3* was reported in 2009 [57]. Since then, few series studying CNVs in HCM patients have been published, and most studies have evaluated only 1 or 2 genes [58–63]. The search for single-exon deletions by long-range PCR in *MYH7* in a cohort of 150 patients did not identify CNVs [58]. Three studies that performed MLPA of *MYBPC3* (and in some cases *TNNT2*) reported a detection rate for CNVs of 0% (0/108) [59], 1% (1/100) [60] and 1.4% (1/72) [61]. Interestingly, the CNV identified in the last two studies was an identical *MYBPC3* large deletion involving several exons (starting in the intron 27 and ending 485 bp after the *MYBPC3* stop codon). Three other cases with CNVs in *MYBPC3* have been reported, but detailed information of each case is not available [62, 63].

The first comprehensive study that searched for CNVs in multiple genes in a large group of HCM patients was published in 2015 by Lopes et al. [23]. They analyzed 19 HCM-related or candidate genes by NGS in 505 patients and detected 4 CNVs (0.8%): 1 deletion in *MYBPC3*, 1 deletion in *PDLIM3*, 1 duplication of the entire *TNNT2* gene, and 1 duplication in *LMNA*. Deletions were considered LP variants, while duplications were considered VUS. Recently, Ceyhan-Birsoy et al. screened 708 HCM patients for CNVs using a NGS panel including 18 HCM-related (or putative) genes or 46 genes covering the full spectrum of cardiomyopathies, and detected CNVs in 4 of them (0.56%): a duplication in *MYOZ2*; a deletion in *MYBPC3*; a whole gene duplication of *NEXN*; and a whole gene duplication of *GLA*, *LAMP2*, *EMD* and *TAZ* (patient with trisomy X) [24]. Only the deletion of *MYBPC3* was classified as pathogenic. In the present study, to further elucidate the role of CNVs in HCM we screened 303 HCM patients for CNVs in 25 genes associated with or candidate for HCM. Among them, we detected 4 CNVs (1.3% of our patients). Two CNVs were novel deletions in *MYBPC3*, one of them involving exon 27 and the other one ranging from exon 4 to exon 12. Both CNVs were classified as pathogenic variants, as radical variants in *MYBPC3* are a well-known cause of HCM [4]. Interestingly, the first patient also harbored one LP variant and one LB variant in *MYBPC3* (p.V771M and p.A522T, respectively), and a VUS in *TTN*. As no family members were available, we were not able to determinate if the *MYBPC3* variants were located in the same allele. To the best of our knowledge, this kind of complex genotype in *MYBPC3* has not been reported before. A previous study in 113 patients designed to search for CNVs in *MYBPC3* in HCM patients carrying one pathogenic point variant did not identify any large rearrangement [61]. Our study demonstrates that even in patients with a LP variant in a main sarcomere gene, the screening for CNVs may add valuable information.

The other two rearrangements identified in our study were deletions of the entire coding region of *PLN* gene. Such *PLN* deletions were classified as pathogenic variants, because the patients only have a single functional copy of the gene, and reduction of the expression of *PLN* (due to nonsense and promoter pathogenic variants) has been previously associated with the development of HCM [64, 65]. These patients did not harbor any other P/LP variant that could explain the HCM phenotype.

The CNV prevalence in our cohort (1.3%) is not significantly different to that reported by Lopes et al. [23] (0.8%, p = 0.4630) and Ceyhan-Birsoy et al. (0.56%, p = 0.2144) [24]. These data suggest that large rearrangements explain a small number of cases that do not carry SNVs and indels, and in selected cases can be part of complex genotypes in combination with variants in sarcomere genes.

### Limitations

Only the protein-coding and flanking intronic regions of known or putative HCM genes were analyzed, and some HCM cases may be explained by pathogenic variants in non-coding regions or other genes. In fact, during the enrollment period of this study new genes that were not included in the cardiomyopathy panels used, such as *FLNC* [66] or *FHL1* [67], have been associated with HCM. Additionally, the inclusion in the HCM panels of genes related to other inherited diseases that involve left ventricular hypertrophy (i.e. Pompe disease, amyloidosis, mitochondrial cardiomyopathies or rasopathies) could also increase the diagnosis of some unexplained cases. The two steps process used for discovery and validation of CNVs showed a high false discovery rate, but this finding might be at least partially related to the inclusion of signals with low quality score in the validation step, in an attempt to minimize the rate of false negatives in a clinical scenario. Even using this low threshold, the number of CNV signals identified is small and their validation does not significantly impact the total cost of genetic testing in the whole sample.

## Conclusion

Only a small percentage of HCM cases without point mutations in the 5 principal sarcomere genes are explained by pathogenic variants in minor or candidate genes for HCM or CNVs, but their identification is of major clinical relevance and can be easily performed by widely available NGS techniques. Screening for *TTN* in HCM patients drastically increases the number of inconclusive tests, and provides a rate of rare variants similar to that found in patients without structural heart disease, suggesting that this gene should not be analyzed for clinical purposes in HCM patients.

## Supporting information

S1 Table Isoforms analysed of the 25 known or candidate HCM genes included in the custom NGS panels. (XLSX)

S2 Table Genetic variants with ExAC MAF <0.002 identifed in 387 consecutive unrelated Spanish patients with hypertrophic cardiomyopathy. (XLSX)

S3 Table Rare variants (MAF <0.0001) in the 5 most frequent sarcomere genes, 25 genes associated with or candidates for HCM and 24 genes (same panel excluding *TTN*). (XLSX)

S4 Table Nonsynonymous variants in *TTN* with ExAC MAF <0.002 identifed in 427 consecutive unrelated Spanish patients without structural heart disease. (XLSX)
